# Analysis of the PDZ binding specificities of Influenza A Virus NS1 proteins

**DOI:** 10.1186/1743-422X-8-25

**Published:** 2011-01-19

**Authors:** Miranda Thomas, Christian Kranjec, Kazunori Nagasaka, Greg Matlashewski, Lawrence Banks

**Affiliations:** 1 International Centre for Genetic Engineering and Biotechnology, Padriciano 99, 34012 Trieste, Italy; 2 McGill University, Montreal, Canada; 3 Department of Obstetrics and Gynecology, Graduate School of Medicine, University of Tokyo, Tokyo, Japan; 4 World Health Organization, Avenue Appia 20, 1211 Geneva 27, Switzerland

## Abstract

The Influenza A virus non-structural protein 1 (NS1) is a multifunctional virulence factor with several protein-protein interaction domains, involved in preventing apoptosis of the infected cell and in evading the interferon response. In addition, the majority of influenza A virus NS1 proteins have a class I PDZ-binding motif at the C-terminus, and this itself has been shown to be a virulence determinant.

In the majority of human influenza NS1 proteins the consensus motif is RSxV: in avian NS1 it is ESxV. Of the few human strains that have the avian motif, all were from very high mortality outbreaks of the disease. Previous work has shown that minor differences in PDZ-binding motifs can have major effects on the spectrum of cellular proteins targeted. In this study we analyse the effect of these differences upon the binding of Influenza A virus NS1 protein to a range of cellular proteins involved in polarity and signal transduction.

## Introduction

The Influenza A virus NS1 protein (non-structural protein 1) is extremely important in the pathology of the virus. It is not a virion component, but is expressed early in infection. It is a multifunctional virulence factor and many of its effects are modulated by activation of PI3K, which it binds via its SH3 domain [1-4].

The influenza A virus NS1 protein has several protein interaction sites, including SH2 and SH3 domains, as well as recognition sites for kinases, including CK2 and MAPK. In addition, over 99% of NS1 proteins isolated have a class 1 PDZ binding motif (PBM) at the C-terminus [5]. PDZ domains are 80-90 amino acid domains that function as docking regions for protein-protein interactions [6,7], and PDZ-containing proteins were originally thought mainly to act as scaffolding proteins for bringing other proteins in proximity to one another, often at the cell membrane. They are now thought to play a more dynamic role, having various functions in cell polarity and cell signalling, depending upon cell cycle and cellular location of the protein (for overviews see Oncogene (2008) **27**, review issue 55).

The importance of the PDZ binding motif (PBM) for influenza virulence was suggested by studies finding, in some cases, that attenuated virulence correlated with C-terminal truncations or extensions of the NS1 protein, either deleting or masking the PBM [8-10]. The avian influenza NS1 protein has recently been shown to interact with a number of PDZ domain-containing proteins including MAGI-1,-2, and -3, Dlg and Scribble [11]. Furthermore, NS1's targeting of Scribble has been shown to relocalise it, concomitantly reducing Scribble-induced apoptosis in infected cells.

We have previously shown that the precise amino acid residues composing the PBM are extremely important in substrate selection [12,13] and we were therefore interested in analysing these differences between the avian-like and human-like PBMs.

## Materials and methods

### Plasmids

The pCDNA 3.1 plasmids expressing human and avian wild type NS1 proteins have been described previously [5] and the Ha, Ah, and Aa mutants were generated in these using the Invitrogen GeneTailor system and verified by sequencing. Oligonucleotides were designed in-house and were synthesised by MWG Biotech AG.

The pCDNA 3.1 plasmids expressing wild type HPV-18 E6 and p53 have been described previously [14].

### *In vitro *translation

The proteins used in this study were translated *in vitro *using the TNT rabbit reticulocyte lysate system (Promega). They were radiolabelled with either [35S]-Cysteine or [35S]-Methionine (Perkin Elmer), depending upon the sequence of the protein in question. The levels of translated proteins were assayed by SDS-PAGE followed by phosphorimager analysis.

### GST pulldown assays

The GST-Dlg, GST-NT Dlg, GST-Dlg N+1 and GST-M1P1 constructs have been described previously [15,16].

The other GST constructs were as follows:

GST-Dlg N+2 expresses Dlg amino acids 1-404;

GST-Dlg N+3 expresses Dlg amino acids 1-539;

GST-M1P5 expresses MAGI-1 amino acids 1034-1115;

GST-NTMAGI expresses MAGI-1 amino acids 1-734;

GST-CTMAGI expresses MAGI-1 amino acids 735-1374;

GST-Scrib4PDZ contains Scrib amino acids 616-1490.

The fusion proteins were immobilised on Glutathione-Agarose (Sigma) and incubated with *in vitro *translated proteins radiolabelled with [35S]-Cysteine or [35S]-Methionine, as described previously [15,16].

### Cells and Transfections

293 cells were maintained in Dulbecco's modified medium supplemented with 10% foetal calf serum, and transfections were performed using the standard calcium phosphate precipitation method [17].

### Interferon induction of STAT1 activation

293 cells were transfected with plasmids expressing human wild type, avian wild type or avian Aa mutant (PDZ non-binding) NS1 proteins or with vector alone. After overnight incubation they were treated with 1 × 10^4 ^U/ml Hplc-purified Interferon-α for 5 h before the total protein extract was analysed by SDS-PAGE and Western Blotting.

### Western blots

Activated STAT1 was detected using anti phospho-STAT1-specific antibodies (Cell Signaling), and α-actinin antibody (Santa Cruz) was used as loading control.

Western blots were developed by the ECL enhanced chemiluminescence method (GE Healthcare) according to the manufacturer's instructions.

## Results

### The human and avian type influenza NS1 proteins differ in PDZ-binding activity

Since the sequence of the NS1 PBM has been shown to affect the virulence of the virus [18], it was of interest to analyse any differences between the PDZ-binding activities of the human and avian NS1 proteins. A PDZ array assay had previously been reported, using a large number of isolated PDZ domains, and this had identified the PDZ domains of several proteins associated with intercellular membranes, including the Dlg PDZ1 domain [5].

**Figure 1:**
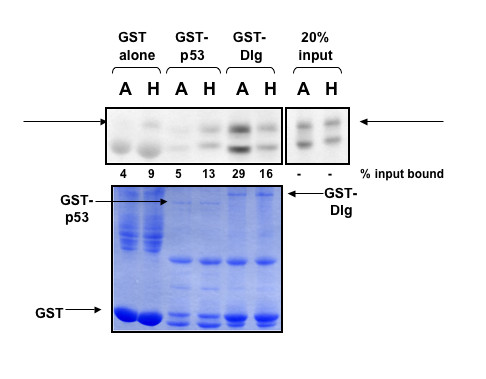
**Human and avian type NS1 protens differ in PDZ-binding activity**. A GST-pulldown assay was performed using bacterially expressed GST and GST-Dlg, with GST-p53 as a non-PDZ-containing control. These were incubated with in vitro translated radiolabelled Avian type (A) or Human type (H) NS1 proteins. After extensive washing the bound proteins were eluted and analysed by SDS-PAGE and autoradiography (upper panel). The lower panel shows the Coomassie-stained gel; the GST fusion proteins are arrowed.

Accordingly, we performed GST pull-down assays, using in vitro-translated, radiolabelled human and avian NS1 protein with bacterially expressed GST and GST-Dlg, using GST-p53 as a non-PDZ protein control. The results are shown in Figure 1; in the upper panel the autoradiograph shows that the avian type NS1 binds markedly more strongly than human type NS1 to GST-Dlg, indicating that the Dlg protein has a much higher affinity for the avian-type ESEV PBM than for the human-type RSKV.

### The NS1 Dlg interaction is PDZ-dependent

**Figure 2:**
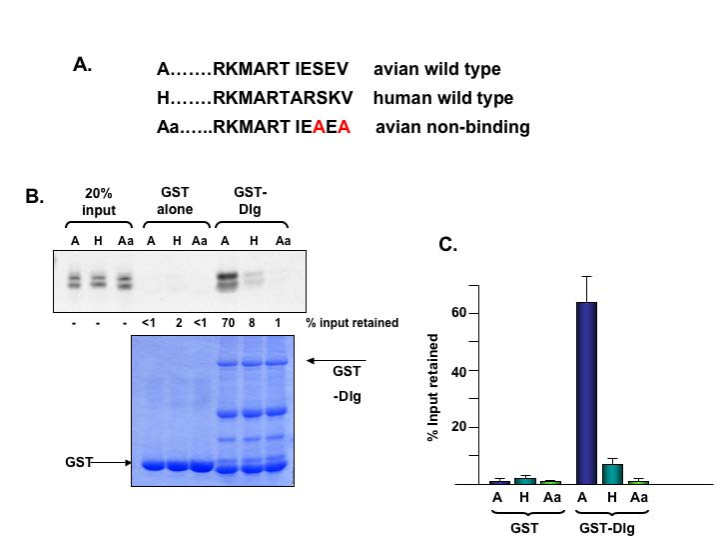
**The NS1 binding to Dlg is PDZ-dependent**. **A**. The cartoon shows the last 11 amino acid residues of Avian (A) and Human (H) NS1, together with the non-PDZ-binding mutant of Avian NS1 (Aa). **B**. GST-pulldown assay, using the NS1 proteins shown in panel A. **C**. Histogram showing the collated results of at least 3 such assays.

To confirm that the NS1 was binding to Dlg through its PBM, the GST pulldown assays were repeated using GST-alone and GST-Dlg, together with in vitro-translated Avian NS1, Human NS1 and a mutant of Avian NS1 in which the C-terminal PBM, ESEV, was mutated to EAEA, thus disrupting its PDZ-binding ability. It can be seen in the upper panel of Figure 2B that the mutant avian NS1 protein (Aa) is indeed defective in binding to the GST-Dlg. The wild type avian NS1 binds strongly and the wild type human NS1 weakly, as seen in Figure 1. The collated results of at least three such assays are shown in Figure 2C.

Thus the interaction between NS1 and Dlg indeed appears to occur primarily through a PDZ-dependent interaction.

### Mapping the the PDZ domain of Dlg targeted by NS1

A number of studies have shown that PDZ-dependent interactions are very specific, with each domain on a multi-PDZ domain protein having specific binding partners [15,19-22]. The human papillomavirus type 18 E6 protein, for example, binds exclusively to Dlg's PDZ2 domain [23]. Thus, having shown that the interaction between NS1 and Dlg was PDZ-dependent, it was interesting to know how selective the influenza A NS1 might be of specific PDZ domain(s) of Dlg.

**Figure 3:**
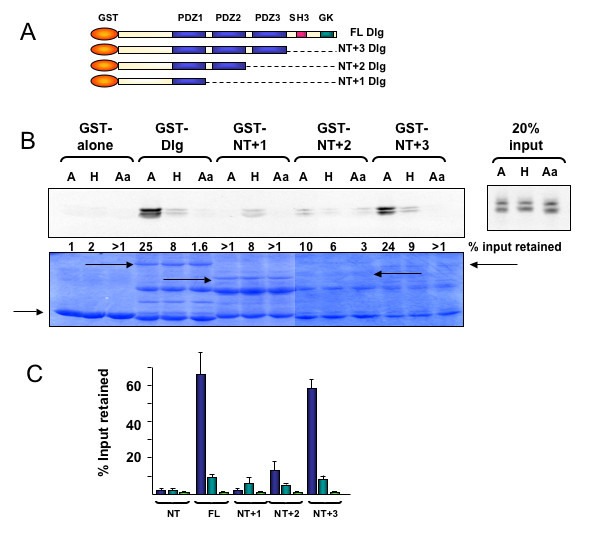
**Mapping the site of NS1 binding on Dlg**. **A**. A cartoon showing the GST-Dlg wild type and mutant fusion proteins used in this assay. **B**. GST pulldown assay, as before, using the mutants shown in 3A. **C**. Histogram showing the collated results of at least three such assays.

To address this question we made use of a panel of Dlg deletion mutants expressed as GST-fusion proteins. Some of these have been described previously [23] but all are shown in Figure 3A for ease of reference. These were used in pull-down assays with the *in vitro *translated avian, human and Aa mutant NS1 proteins. The results of a representative assay are shown in Figure 3B, and a histogram of the collated results from at least three assays are shown in Figure 3C. It is clear from these results that that the major PDZ-dependent binding activity of avian NS1 is directed at the PDZ domain 3. Interestingly, this is in contrast to the results from the PDZ array described by Obenauer and colleagues [5], who identified PDZ1, but not PDZ3 as an NS1-specific target.

### The exact PDZ-binding motif sequence directs the specificity of binding to Dlg

**Figure 4:**
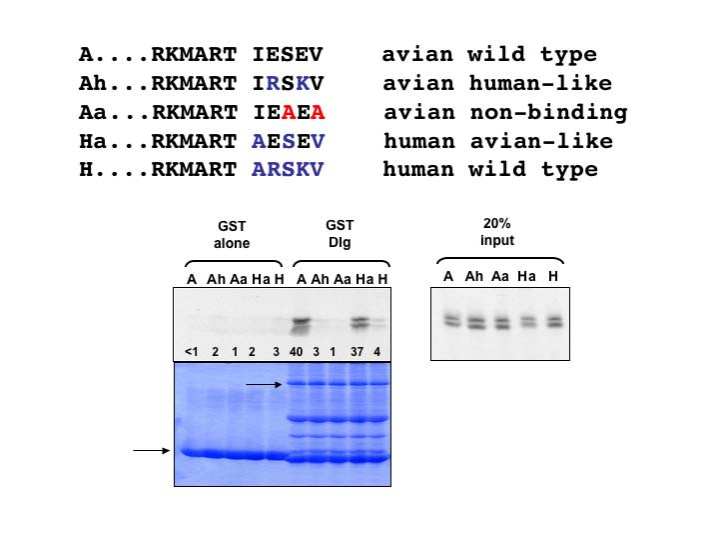
**The non-canonical residues of the PBM determine NS1 binding affinity for Dlg**. **Upper panel**. The cartoon shows the last 11 amino acid residues of Avian (A) and Human (H) NS1, together with the non-PDZ-binding mutant of Avian NS1 (Aa), plus the avian human-like (Ah) and the human avian-like (Ha) mutants. **Lower panel**. GST pulldown assay using these NS1 proteins.

Having shown that the Avian NS1 protein binds to the Dlg PDZ3, we were interested to know exactly what determined the specificity of binding. We had previously shown with human papillomavirus E6 protein that its specificity of binding was related to the presence of specific amino acid residues in and around the PBM [12-14] and it seemed probable that a similar situation would be true for the NS1 protein. To investigate this we introduced specific mutations into the PBM of NS1, and these are shown in Figure 4, upper panel. These were then used in a GST pulldown assay with GST alone and GST-Dlg. It can be seen in Figure 4 (lower panel) that on the GST-Dlg, the binding of the avian type NS1 (A) is almost abolished by substituting the human type residues (Ah), while the very low binding of the human type NS1 (H) is markedly enhanced by substitution of the avian residues (Ha). This result clearly demonstrates that the binding specificity of the NS1 protein to Dlg is determined by the non-canonical amino acid residues within the PBM.

### Sequence requirements for NS1 interactions with the PDZ domains of MAGI-1 and Scribble

Having defined the interaction of NS1 and Dlg PDZ3, it was interesting to know whether similar constraints applied to the binding of NS1 to other PDZ domains.

**Figure 5:**
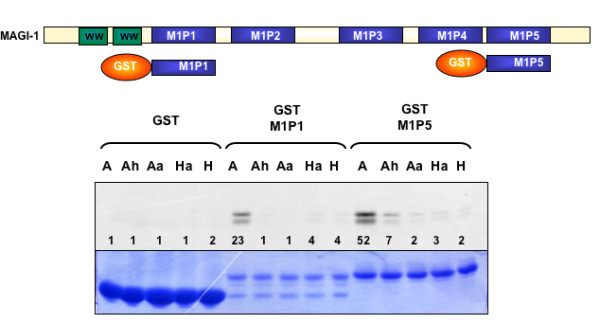
**The avian PBM is not sufficient for binding all class 1 PDZ domains**. **Upper panel**. The cartoon shows the wild type MAGI-1c and the GST fusion proteins M1P1 and M1P5. **Lower Panel**. A GST pulldown assay was performed using these fusion proteins together with the NS1 proteins described in Figure 4.

We performed GST pulldown assays with the PDZ1 (M1P1)and PDZ5 (M1P5) domains of MAGI-1, expressed as GST fusion proteins. It can be seen in Figure 5 that, as with Dlg, the Avian NS1 binds strongly to the GST-M1P1 and GST-M1P5 and the binding is abolished in the Ah mutant. However, the Ha mutant does not bind significantly more than the Human NS1, indicating that the non-canonical residues in the PBM are not sufficient to specify binding, and that probably residues upstream of the PBM may also be involved. This assay, together with the results from mapping the binding to Dlg, raised the question of whether isolated PDZ domains can be used meaningfully in such binding assays, and it also raised a second question: does NS1 really bind to two PDZ domains on the same protein?

**Figure 6:**
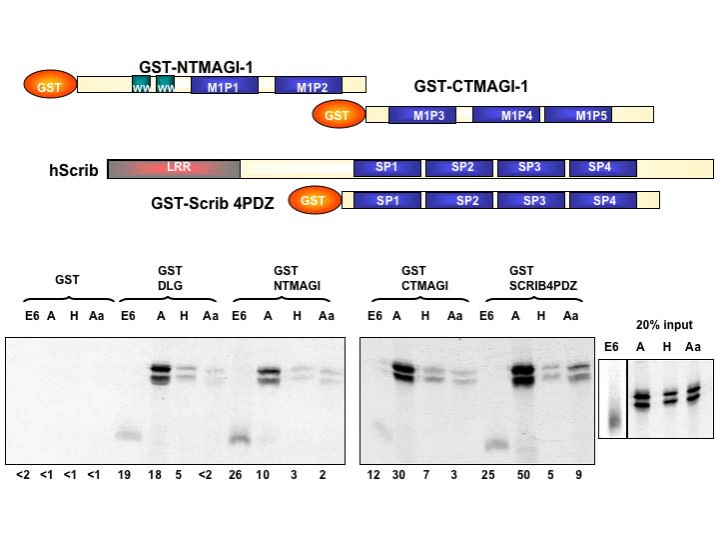
**Different PDZ domains have different binding characteristics for the same ligand**. **Upper panel**. The cartoon shows the GST-NTMAGI-1 and GST-CTMAGI-1 fusion proteins. Also shown is the GST-Scrib4PDZ fusion protein, which comprises all four of the PDZ domains of Scribble, aligned with the full-length protein for reference. **Lower panel**. A GST pulldown assay was performed with these fusion proteins plus the GST alone and GST-DLG as negative and positive controls, respectively. They were incubated as before with the *in vitro *translated Avian, Human and non-PDZ binding mutant NS1 proteins. Human papillomavirus type 18 E6 was included for comparison.

To address these questions we repeated the GST pulldown assays using the two halves of the MAGI-1 protein expressed as GST fusion proteins. In Figure 6 it can be seen that the binding of Avian NS1 to CT-MAGI-1 is markedly stronger than its binding to NT-MAGI-1, in contrast to the human papillomavirus type 18 E6 protein, which binds more strongly to the NT-MAGI-1, consistent with previous data showing that it specifically targets MAGI-1 PDZ1 [20,22,25]. This suggests that the preferred PDZ domain target of NS1 may be M1P5, which would be consistent with the stronger binding seen in Figure 4.

The PDZ domain-containing protein, hScrib, has recently been shown to be a PDZ-dependent target of NS1 [11]. Scrib is a partner of Dlg and of HuGL in the tripartite Scrib complex which contributes to polarity regulation in the cell [see [26,27], for reviews]. As can be seen from Figure 6, the GST-Scrib4PDZ is bound very strongly by the avian NS1, and weakly by the human NS1; but most interestingly the Aa mutant, which has a non-functional PBM, still binds more strongly to GST-Scrib4PDZ than the human wild type, albeit much less than the wild type avian. This indicates that the NS1 protein interaction with Scrib is mainly mediated by the PBM, but that other regions of the protein may also be involved.

### NS1 effect upon hScrib's signalling activity

**Figure 7:**
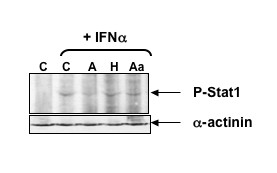
**Expression of wild type avian NS1 reduces Interferon-induced STAT1 activation**. **Upper panel**. Western blot analysis of 293 cells transfected with plasmids expressing wild type avian NS1 (A), wild type human NS1 (H), non-PDZ binding avian NS1 mutant (Aa) or vector alone (C). Cells were treated for 5 h with 1 × 10^4 ^U/ml Hplc-purified Interferon-α prior to harvesting. The blot was probed with anti phospho-STAT-1 antibodies to detect activated STAT1. **Lower panel**. The blot was reprobed with anti-α-actinin antibody to control for cellular protein input.

Having shown that NS1 binds strongly to hScrib, it was interesting to know how that might contribute to the viral life cycle. C-terminal truncations of NS1 had been associated with increase in interferon (IFN) activity via JAK/STAT signalling [28; 29], and the Tick-borne encephalitis virus (TBEV) NS5 protein has been shown to impair IFN-stimulated JAK/STAT signalling in an hScrib-dependent manner [30]. We were therefore interested to know whether the ability of influenza A NS1 to alter JAK/STAT signalling required PDZ-binding activity. To investigate this, we transfected 293 cells with plasmids expressing the Avian, Human, or Aa mutant NS1 proteins, treated them with purified IFNα and analysed the STAT activation by Western blot. As can be seen in Figure 7, IFNα strongly induces the phosphorylation of STAT, and this is markedly reduced in the presence of Avian NS1 and to a lesser extent in the presence of Human NS1 or the Aa mutant. This indicates that the ability of NS1 to bind to PDZ substrates correlates with its ability to reduce STAT activation.

## Discussion

In this study we have dissected the PDZ binding activities of the Avian and Human type NS1 proteins of Influenza A. It is clear from these studies that the Avian type PBM binds PDZ domains more strongly than does the Human type PBM. It also shows that there are interesting differences in their modes of interaction, depending upon the PDZ domain analysed.

Previous work has shown that screening for protein interactions using isolated PDZ domains can be misleading in determining which PDZ domains are the true binding partners of certain proteins. Our assays using the full-length Dlg protein show differences from data published using only isolated domains (5). In addition, our assays shown in Figures 5 and 6 using either the isolated PDZ domains of MAGI-1 or larger portions of the protein would tend to suggest that data obtained from single domain assays should be treated with caution.

We had previously shown by crystallographic and mutational analysis that the specificities of type 1 PDZ-binding interactions are determined by several factors [12,13]. The sequence of the canonical motif: x-S/T-x-V/L/I is highly influential and we have shown that changing the V to L in otherwise identical PBMs alters target selection [22,24]. Furthermore, the presence of serine or threonine can affect target selection, even between highly homologous PDZ domains, depending on the hydrophobicity of the PDZ domain's binding groove [13]. The third layer of selectivity is contributed by the non-canonical -4 and -2 amino acid residues (numbering the final residue as -1), and by the residues immediately upstream of the PBM. We have shown that the influence of these residues can be critical in determining PDZ domain preference, and hence substrate selectivity [12,13].

In this study we have shown that the Avian type NS1 protein binds strongly to the Dlg PDZ3 domain and it might be reasonable to speculate that type 1 PDZ domains of similar sequence might also be targeted by Avian NS1. We have shown that the binding is specific, and this supports the data of Liu et al [11] who showed the binding between GST-NS1 and HA-tagged Dlg exogenously expressed in 293T cells. They also showed that the binding of NS1 to each of the highly homologous MAGI-1,-2 and -3 proteins is not equally strong, again supporting our findings that the binding selectivity is mutually determined by the sequences of both PDZ domain and ligand.

As seen in Figure 6 the avian NS1 binds to hScrib, largely through PDZ interactions and this agrees with the data of Liu et al., [11]. The absence of a PBM in truncated NS1 proteins increases the expression of IFN [29] and correlates with attenuated virulence [8-10]. This, together with the finding that TBEV NS5 binds hScrib and impairs IFN-stimulated JAK/STAT signalling, possibly through feedback between STAT and IFN [30], led us to investigate the effect of a functional PBM upon IFN-induced STAT activation. Our results show that STAT phosphorylation induced by IFNα is reduced in the presence of an hScrib-binding PBM, and is essentially unaffected by the same protein with two point mutations that render the PBM inactive. Clearly, other PDZ domain proteins could also be involved in this activity, although hScrib is a strong candidate, based upon its strength of interaction and previous studies linking hScrib to the regulation of STAT signalling. It seems possible that this function of NS1 is to assist the virus in evading the IFN response to infection. Soubies et al [29] showed that truncation of NS1 increases IFN induction during infection and Zielecki et al. [31] have shown that the presence of an avian type PDZ motif can modulate viral replication in a strain and host-dependent manner. These studies underline the importance of the PDZ domain and support the notion that the strength of PDZ interactions is mediated by the precise sequences of the PDZ domain in question and the canonical and non-canonical residues composing and upstream of the PDZ binding motif.

## Competing interests

The authors declare that they have no competing interests.

## Authors' contributions

MT participated in the conception and design of the study, carried out the binding assays and drafted the manuscript. CK constructed the MAGI-1 fusion proteins. KN constructed the hScrib fusion proteins. GM participated in the conception of the study. LB participated in the conception and design of the study and performed the western blots. All authors read and approved the final manuscript.
